# Repertory of Diptheria

**Published:** 1893-04

**Authors:** Wm. Jefferson Guernsey

**Affiliations:** Philadelphia


### REPERTORY OF DIPHTHERIA,

REVISED AND ENLARGED.

BY WM. JEFFERSON GUERNSEY, M. D., PHILADELPHIA.

### SUBJECTIVE.

ACHING. (Painful, simple, pressive, ordinary, dull, indescribable, undefined pain. Anguish, misery, suffering, torture, agony.) Acon., Æscul., Am-cb., Am-caust., *Apis*, Arsen., Bapt., *Bell.*, Bryon., Calc-ph., Canth., Caps., Carbol-ac., *Cistus*, Hepar, Ign., Iod., Kal-perm., *Lach.*, Lac-can., Lyc., Merc., Merc-cor., Merc-j-fl., Merc-j-ru., Nit-ac., Phytol., Rhus, Sal-ac., Sul.

BORING. (Digging, rooting, gnawing.) Alum., Arg-met., Lac-can., Paul.

BURNING. (Heat, warmth, scalding.) Acon., *Æscul.*, Am-cb., Arsen., Arum, Bell., Canth., Caps., Lac-can., Merc., Merc-cor., Nit-ac., Sal-ac., Sang., Sinap., Sul.

BURNING LUMP. (As of a hot ball.) *Phyt.*

BURNING, STINGING. *Apis.*

BURNT, as if. See SMARTING.

BURSTING, as if. See FULLNESS.

CHAFED, as if. See SMARTING.

CLOSED, as if. See SQUEEZING.

CLUTCHING, as of. See SQUEEZING.

COMPRESSION, as of. See SQUEEZING.

CONGESTION, as of. See FULLNESS.

CONSTRICTION, as of. See SQUEEZING.

CONTRACTION, as of. See SQUEEZING.

CONTUSED feeling. See SORENESS.

CUTTING. (Acute pain when undefined. Darting pain, knife thrusts, piercing, penetrating pain, severe pain, sharp pain, shooting pain, lancinating, neuralgic pain, long stitches.) Æscul., Am-caust., Arsen., Igna., Lac-can., Bell., Kali-nit., Sul.

DARTING. See CUTTING.

DIGGING. See BORING.

DISTENTION. See FULLNESS.

DRAGGING PAIN. See ACHING.

DRAWING PAIN. See ACHING.

DRAWING SENSATION. See TENSION.

DRYNESS, sensation of. Acon., Æscul., Ailanth., *Alum.*, Apis, Arg-nit., Arsen., Arum, *Bell.*, Bryon., *Cistus*, Ign., Kali-bi., Kali-perm., *Lac-can.*, Lyc., *Merc.*, Merc-cor., Phyt., Sang., Sinap., Sticta, *Sul.*

DULL PAIN. See ACHING.

ERODED sensation. See SMARTING.

EXCORIATED sensation. See SMARTING.

EXPANDING, as if. See FULLNESS.

FOREIGN BODY, sensation as of a blunt. See LUMP.

FRICTION, sensation of. See SCRAPING.

**SUBJECTIVE**—*Continued.*

FULLNESS, feeling of. (Congested, as if; bursting, as if; pressed asunder, as if; thick feeling, expanding sensation, swollen sensation, tumid feeling, bloated sensation, large, as if; distended sensation.) Ailanth., Alum., *Apis*, Bapt., Bell., Brom., Lac-can., Lach., Lact-ac., *Phyt.*, Puls.

GNAWING. See BORING.

GRATING. See SCRAPING.

HAIR, as from a. See TICKLING, etc.

KNIFE THRUSTS. See CUTTING.

LANCINATING. See CUTTING.

LARGE, as if. See FULLNESS.

LUMP, as of a. (Ball, plug, foreign, blunt body.) Æscul., Alum., Am-carb., Apis, Bell., Hepar, *Igna.*, Kali-bi., LAC-CAN., *Lach.*, Merc-j-fl., Merc-j-ru., Merc-viv., Phyt., Sul.

LUMP, HOT. See BURNING LUMP.

NEURALGIC PAIN. See CUTTING.

NUMBNESS. *Acon.*

PAIN, DULL. See ACHING.

PAIN, SEVERE. See CUTTING.

PAIN, WITHOUT, although the throat is inflamed. *Apis.*

PEPPER, as from. See SMARTING, etc.

PLUG. See LUMP.

PRESSING ASUNDER. See FULLNESS.

PRESSIVE PAIN. See ACHING.

PRICKING. See STICKING.

PULLING. See TENSION.

PULSATION. See THROBBING.

PUNGENT. See SMARTING.

RASPING. See SCRAPING.

RAWNESS. See SMARTING.

ROUGHNESS. Ailanth., Sul., Sul-ac.

SCALDED. See SMARTING.

SCALDING. See BURNING.

SCRAPING. (Grating, rubbing, friction, rasping.) Acon., Ailanth., Arsen., Bell., Bryon., Hepar, Kali-bi., Nit-ac., Sul.

SENSITIVENESS. See AGG. TOUCH.

SEVERE PAIN. See CUTTING.

SHARP PAIN. See CUTTING.

SMARTING. (Excoriation; burnt, as if; scalded, corrosive, eroded, ulcerated, as if; acrid, chafed, raw, biting, pungent.) Arum., Lach., *Lac-can.*, Merc-j-ru., Nit-ac., Phyt.

SMARTING, PEPPER, as from. Caps.

SMARTING SPOT. Mur-ac.

SORENESS. (Contused, as if; bruised, as if.) Ailanth., Alum., Apis, Arsen., Arum., Bell., Bryon., Caps., LAC-CAN., *Lach.*, Lyc., Merc-cor., Merc-j-ru., Merc-viv., Mur-ac., Nit-ac., Phytol., Sul.

### SUBJECTIVE—*Continued.*

SORENESS, SPOTS OF. Bary-cb.

SPOTS OF SMARTING. See SMARTING SPOT.

SPOTS OF SORENESS. See SORENESS, SPOTS OF.

SQUEEZING. (Constriction, compression, bound feeling, closed, grasped, contracted, clutched.) Æscul., Ailanth., Alum., Apis, Bapt., *Bell.*, Brom., Canth., Caps., Chel., *Hepar*, Igna., LAC-CAN., *Lach.*, Lyc., Phyt.

STICKING. (Pricking.) Acon., Æscul., Alum., Apis, Bell., Bry., Hepar, *Ign.*, Kali-bi., Lac-can., Lach., Lyc., Merc-j-ru., Merc-viv., Nit-ac., Sul.

STINGING, BURNING. See BURNING, STINGING.

SWOLLEN, as if. See FULLNESS.

TENDERNESS. See AGG. TOUCH.

TENSION. Arg-nit.

THICK FEELING. See FULLNESS.

THROBBING. Am-mur., *Arg-nit.*, *Bell.*, Chel., Merc., Rhus.

TICKLING HAIR, as from a. Arsen., Kali-bi., Sil.

TIGHTNESS. See TENSION.

ULCERATED, as if. See SMARTING.

### OBJECTIVE.

BLUISH. *Ailanth.*, Am-carb., Kali-bi., Lach., Merc-cyan., Phytol., Sul.

BRIGHT RED. See INFLAMED, BRIGHT RED.

BROWNISH. See INFLAMED, BROWNISH.

CONGESTED. See INFLAMED.

DARK RED. See INFLAMED, DARK.

DISCHARGE, ACRID. Alum., Arsen., *Arum.*, Merc-viv.

DISCHARGE, BLOODY. Apis, Arsen., Arum, Merc-viv., *Rhus*, Sul-ac.

DISCHARGE, EXCESSIVE. Apis, Canth., Iod., Lac-can., Lach., Merc-cyan., Merc-viv., Nit-ac., Sul.

DISCHARGE, FETID. See FETID.

DISCHARGE, VISCID. Kali-bi., Lach., Merc-j-fl.

DRYNESS. Acon., Arsen., Bryon., Lach., Merc-viv., Sticta, *Sul.*

ERYSIPELATOUS, swelling. See SWELLING, ERYSIPELATOUS.

EXCORIATED, as if. See INFLAMED, EXCORIATED, AS IF.

FETID. (Including the offensive odor of discharge, membrane or breath.) Ailanth., Alum., Am-carb., Apis, Arsen., Arsen-jod., Arum, Bapt., *Carbol-ac.*, Iod., Kali-bi., Kali-perm., Lac-can., Lach., Lyc., *Merc-cyan.*, Merc-j-fl., *Merc-viv.*, *Nit-ac.*, Phytol., Sul-ac.

FOUL SMELLING. See FETID.

GANGRENE. Am-cb., Arsen., Lach., Merc-cor., Merc-cyan., Sul.

GANGRENE, LATER STAGES. Arsen.

GANGRENE, RAPIDLY DEVELOPED. Lach.

GANGRENE, THREATENED. Arsen., Lach.

GATHERING. See SUPPURATING.

INFLAMED. Acon., Ailanth., Am-caust., *Arg-nit.*, Bapt., *Bell.*, Canth., Igna., Kali-bi., Lac-can., Lach., Lyc., Merc-cor., Merc-cyan., Merc-j-fl., Merc-p-rub., Merc-viv., Mur-ac., Nit-ac., Phytol.

### OBJECTIVE—*Continued.*

INFLAMED, BRIGHT RED. Acon., Apis., *Bell.*
INFLAMED, BROWNISH. Lyc.
INFLAMED, DARK RED. *Æscul.*, Ailanth., Bapt., Lac-can., Merc-j-ru., ***Phytol.***
INFLAMED, EXCORIATED, as if. Arum.
INFLAMED, INTENSELY. *Apis.*
INFLAMED, SHINING. Bell., Lac-can.
INFLAMED, SLIGHTLY. Carbol-ac., Lac-can.
IRRITATED, looking. See INFLAMED.
LIVID. See BLUISH.
MEMBRANE, ADHERING firmly. Kali-b.
MEMBRANE, BLOOD STREAKED. Kali-b.
MEMBRANE, BLUISH. Carbol-ac., Lach., Merc-cyan., **Merc-j-ru.**
MEMBRANE, BROWNISH. Iod., Lac-can.
MEMBRANE, CURDY. Lac-can.
MEMBRANE, DARK. Bapt., Lac-can., Phytol.
MEMBRANE, DEEP SEATED. Ailanth., Apis, Kali-bi., **Nit-ac.**
MEMBRANE, DIRTY looking. Apis, ***Lac-can.***
MEMBRANE, DRY. Arsen.
MEMBRANE, DRY, leather like. Sticta.
MEMBRANE, ELASTIC. Kali-bi.
MEMBRANE, FETID. See FETID.
MEMBRANE, FIBRINOUS. Kali-bi.
MEMBRANE, GRAY. Apis, Carbol-ac., Iod., Kali-bi., **Lac-can.**, **Lyc.**, **Merc-cyan.**, Merc-j-fl., Mur-ac., Nit-ac., Phyt., Sul-ac.
MEMBRANE, GREENISH. Kali-bi., Lac-can.
MEMBRANE, IRREGULAR shaped. Lac-can., Merc-j-fl.
MEMBRANE, ISLANDS OF. See MEMBRANE, PATCHES.
MEMBRANE, LEATHERY. Merc-cyan.
MEMBRANE, LOOSE. Lac-can., Merc-j-fl., Merc-j-ru.
MEMBRANE, MARGIN ADVANCING. Apis.
MEMBRANE, MIGRATORY. Lac-can.
MEMBRANE, OFFENSIVE. See FETID.
MEMBRANE, PALE. Sinap.
MEMBRANE, PATCHES, in. Canth., Merc-j-ru.
MEMBRANE, PATCHES, SMALL. *Ailanth.*, Canth., Igna., Iod., Kali-bi., LAC-CAN., *Merc-j-fl.*, Merc-j-ru.
MEMBRANE, PEARLY. Kali-bi., *Lac-can.*, Sang.
MEMBRANE, PROFUSE. Carbol-ac., Lach., Merc-cor., Sul-ac.
MEMBRANE, PUTRID. See FETID.
MEMBRANE, RED MARGIN. Apis.
MEMBRANE, REMAINS LONG. *Sul.*
MEMBRANE, SCANT. Apis, Lac can., Merc-j-fl., Merc-j-ru.
MEMBRANE, SHINY MARGIN. Apis.
MEMBRANE, SOFT. Sul-ac.
MEMBRANE, SPONGY. Alum.

### OBJECTIVE—*Continued.*

MEMBRANE, THICK. Arsen., Iod., Lac-can., Sul-ac.
MEMBRANE, THIN. Lac-can., Merc-cyan.
MEMBRANE, TRANSPARENT. Merc-j-fl., Merc-j-ru.
MEMBRANE, VARIEGATED. *Lac-can*
MEMBRANE, VARNISHED looking. Lac-can.
MEMBRANE, WASH-LEATHER like. Bapt.
MEMBRANE, WHITE. Am-caust., Iod., Lac-can., Lachanth., Lyc., Merc-cyan., Merc-j-fl., Mur-ac., Nit-ac.
MEMBRANE, WRINKLED looking. Arsen.
MEMBRANE, YELLOWISH. Kali-bi., Lac-can., Lach., Merc-cyan., Nit-ac., Rhus, Sul., Sul-ac.
ŒDEMA. See SWELLING, ŒDEMATOUS.
OFFENSIVE. See FETID.
PURPLE. See BLUISH.
PUTRID swelling. See FETID.
RAW. See INFLAMED, EXCORIATED, as if.
REDNESS. See INFLAMED.
SHINING. See INFLAMED, SHINING.
SMELLING, badly. See FETID.
STINKING. See FETID.
SUPPURATING. *Bary-cb.*, *Hep.*, Kali-bi., Lac-can., Lach., Lyc., Merc-cor., Merc-j-fl., Merc-j-ru., *Merc-viv.*, Niccol., Nit-ac., *Phytol.*, *Sil.*, Sul.
SWELLING. *Æscul.*, *Ailanth.*, Am-cb., Apis, Arsen., Arum., Bapt., *Bary-cb.*, *Bell.*, Bryon., Canth., Hepar, Igna., Kali-bi., *Lac-can.*, *Lach.*, *Lyc.*, Merc-cor., Merc-j-fl., Merc-j-ru., Merc-viv., Mur-ac., Nit-ac., *Phytol.*, Sal-ac., Sul-ac.
SWELLING, ERYSIPELATOUS. *Apis*, Bell., Merc., Rhus.
SWELLING, ŒDEMATOUS. Ailanth., Am-carb., Apis, Bapt., *Kali-bi.*, Kali-chlor., Kali-iod., Mur-ac., Nit-ac., Rhus, Sul-ac.
VARNISHED LOOKING. Lac-can.
VESICLES. Apis.

### AGGRAVATION AND AMELIORATION.

AIR, COLD. See COLD AIR.
BENDING HEAD BACK. Aggr. Lach.
BEGINNING to swallow. See SWALLOW, etc.
BREATHING. Aggr. Hep.
BREATHING cold air. See COLD AIR.
BREATHING IN. Agg. Ailanth., Apis, Arum.
CANDY. See SWEETS.
COLD AIR. Agg. Cistus, Merc.
COLD AIR. Amel. Sang.
COLD DRINK. Aggr. Canth., *Lyc.*
COLD DRINK. Amel. Lac-can.

### AGGRAVATION AND AMELIORATION—*Continued.*

COLD FOOD. Aggr. Lyc.
COMMENCING SWALLOWING. See SWALLOWING, etc.
COUGHING. Aggr., Acon., Caps., Hep.
DRINKS, COLD. See COLD DRINK.
DRINKS, SWALLOWING. See SWALLOWING, etc.
DRINKS, WARM. See WARM DRINKS.
FOOD, COLD. See COLD FOOD.
FOOD, SWALLOWING. See SWALLOWING, etc.
FOOD, WARM. See WARM FOOD.
GAPING. Agg. Arg-met., Niccol.
HEAD, BENDING BACK. See BENDING, etc.
HEAD TURNING. See TURNING.
HOT THINGS. See WARM.
INHALING. See BREATHING IN.
LYING. Aggr. Bell.
MOVING TONGUE. Aggr. Ambr.
NECK, TURNING. See TURNING.
PROTRUDING TONGUE. Agg. Kali-bi.
RESPIRATION. See BREATHING.
ROOM, WARM. See WARM ROOM.
SALIVA, SWALLOWING. See SWALLOWING, etc.
SLEEP, AFTER. Aggr. Kali-bi., Lach., Lac-can., Merc-j-ru.
SPEAKING. Aggr. Acon., Bell., Merc., Rhus.
SUDDEN. Aggr. or Amel. Bell.
SUGAR. See SWEETS.
SWALLOWING. Aggr. *Acon.*, *Æscul.*, Ailanth., Aloe, Anac., APIS, Arg-met., *Arsen.*, Arum., Asaf., *Bary-cb.*, BELL., Bovist., *Brom.*, BRYON. Calc., Calc-ph., Camph., *Canth.*, Caps., Cham., China, Cinnab., *Coccul.*, Coff., Colch., Con., *Crocus.*, Crotal-hor., Croton-tig., Dros., Elaps., *Gels.*, Hell., HEP., Hydras., *Hyos.*, Igna., *Iod.*, Kali-bi., Kali-bro., Kali-cb., Kali-chl., Kali-nit., Kreos., LACH., LAC-CAN., *Lyc.*, Mag-cb., Meny., *Merc-cor.*, MERC-J-FL., Merc-j-ru., MERC-VIV., *Mur-ac.*, Nat-mur., NIT-AC., Nux, Opi., Petrol., Phos., Physos., *Phytol.*, Plat., Plumb., *Pul.*, Ran-bulb., Rhod., *Rhus*, Ruta, Sang., Sars., Sep., Sil., Spig., Stan., Staph., *Stram.*, SUL., *Sul-ac.*, Tart., *Thu.*, Verat.
SWALLOWING. Amel. Alum., *Ambr.*, *Arg-met.*, *Arn.*, Bapt., Bell., *Caps.*, Chel., Cistus, Dig., Graph., IGNA., Kali-bi., *Lach.*, Lac-can., Laur., Mang., Merc., Mezer., *Nit-ac.*, *Nux*, Paris, Phos-ac., Puls., Rheum, Sabadil., Sabina, *Spong.*, Squil., Stan., Staph., Sul., Sul-ac., Tarax., Zinc.
SWALLOWING, BEGINNING. Aggr. Rhus.
SWALLOWING, COLD. See COLD, etc.
SWALLOWING DRINKS. Amel. Lac-can.
SWALLOWING, EMPTY. See SWALLOWING SALIVA.
SWALLOWING FOOD, AFTER. Agg. *Ambr.*, Bary-c., *Bryon.*, Hep., Iod., Nit-ac., *Nux*, Petrol, *Phos.*, *Puls.*, *Rhus*, Sep., *Sul.*, Zinc.

### AGGRAVATION AND AMELIORATION—*Continued.*

SWALLOWING FOOD, DURING. Agg. Alum., Bapt., *Bary-cb.*, *Bry.*, *Hep.*, Iod., Lach., Lac-can., Nit-ac., *Nux*, Petrol., Phos., Phos-ac., *Rhus*, Sep., *Sul.*, Zinc.

SWALLOWING FOOD, DURING. Amel. *Ign.*

SWALLOWING LIQUIDS. Aggr. BELL., Bry., *Canth.*, Cina, Hyos., Igna., Iod., LACH., Lyc., *Merc-cor.*, *Merc-viv.*, Phos., *Stram.*, Sul-ac.

SWALLOWING SALIVA. Aggr. Arg-met., *Bary-cb.*, Bell., *Bry.*, Caps., Crocus, Hepar, LACH., Lac-can., Merc-j-fl., Merc-j-ru., Merc-viv., Nux, Puls., Rhus, Sul., Thu.

SWALLOWING WARM things. See WARM.

SWEETS. Aggr. Sang.

SWEETS. Amel. Arsen., Bell.

TALKING. See SPEAKING.

TONGUE, MOVING. See MOVING TONGUE.

TONGUE, PROTRUDING. See PROTRUDING TONGUE.

TOUCHING neck. Aggr. Ailanth., *Apis*, Bell., Bry., Kali-bi., LACH., *Lac-can.*, Phytol., Sal-acid.

TOUCHING NECK, LEFT side. Aggr. *Lach.*

TOUCHING NECK, RIGHT side. Aggr. *Niccol.*

TURNING HEAD. Aggr. Bell., Brom., Bry., *Hepar*, Lach.

TWISTING NECK. See TURNING HEAD.

WARM DRINKS. Aggr. *Lach.*, Phytol.

WARM DRINKS. Amel. *Lyc.*

WARM FOOD. Amel. *Lyc.*

WARM ROOM. Aggr. Bry.

WARMTH in general. Amel. Arsen.

WATER, DRINKING COLD. See COLD DRINKS.

YAWNING. See GAPING.

### REGION.

**Affected either Subjectively or Objectively.**

ARCH OF PALATE. See PALATE, ARCH OF.

BACK OF FAUCES. See FAUCES, POSTERIOR.

BEGINNING ON. See part affected.

DOWNWARD, EXTENDING. Brom., Canth., Lac-can.

EARS, EXTENDING TO. Apis, Bell., Calc., Gels., *Hepar*, Igna., Ipec., *Kali-bi.*, Kali-nit., Lach., *Lac-can.*, Mag-mur., Merc., Nux, *Phytol.*, Sal-acid, Sars., Sul.

EARS, EXTENDING TO LEFT. *Kali-bi.*, *Lac-can.*

EARS, EXTENDING TO RIGHT. Brom., Lac-can., Sol-nig.

ENTIRE THROAT. Arsen., Am-caust., Kali-perm., Lac-can., Sul.

EXTENDING TO OF FROM. See part affected.

FAUCES, LEFT SIDE. See SIDE, LEFT.

FAUCES, POSTERIOR. Æscul., Apis, Am-caust., Canth., Kali-bi., *Lac-can.*, *Merc-j-fl.*, Mur-ac., Nit-ac., Phytol., *Sul.*

### REGION—*Continued.*

FAUCES, RIGHT SIDE. See SIDE, RIGHT.

GUMS, extending to. Lach.

LARYNX. Acon., *All-cep.*, Am-mur., Apis, Arg-met., Arg-nit., Arsen., Arsen-jod., Arum, Bapt., Bary-cb., *Bell.*, *Brom.*, Callad., Canth., Caps., Carb-veg., Caust., Cham., Cimicif., Cina, Coccul., Coff., *Dros.*, *Hep.*, Iod., Ipec., *Kali-bi.*, Kali-iod., *Kali-perm.*, *Lach.*, *Lac-can.*, Lactuc., Laur., Mang., *Nux*, Osmium, Paris, *Phos.*, *Puls.*, Sabadil., Seneg., *Spong.*, Sul., Verat.

LARYNX, BEGINNING IN. Brom., Lac-can.

LARYNX, EXTENDING TO. Brom., Lac-can., Lach.

LEFT SIDE. See SIDE, LEFT.

LIP. *Arsen-jod.*, *Arum*, Bell., Bryon., Na-mur., Rhus, Sepia, Sul.

LIP, LOWER. *Bry.*, Calc., Ign., Na-cb., Na-mur., Puls., Sep., *Sul.*

LIP, UPPER. *Arsen.*, Bary-cb., Bell., Carb-veg., Caust., Cicuta, Graph., *Kali cb.*, Kreos., Merc-viv., Na-cb., Na-mur., Psor., Rhus, Staph., *Sul.*

MOUTH. Acon., Alum, ARSEN., *Arum*, Bapt., Bary-cb., BELL., Borax, Bry., Calc-cb., Canth., Caps., Carb-veg., Cham., China, Flu-ac., Igna., Iod., *Kali-bi.*, KALI-CHL., *Lach.*, Lyc., *Merc-cor.*, Merc-cyan., MERC-VIV., Mezer., Mur-ac., Nit-ac., *Nux*, *Phos.*, Puls., Rhus, Sabadil., Sep., Stram., *Sul.*, Sul-ac., Thu.

MOUTH, AROUND. *Agar.*, Aloe, Alum., Am-cb., Ant-crud., ARSEN., ARUM, Bary-cb., Bell., *Bovis.*, BRYON., *Calc.*, Can-sat., Caps., *Caust.*, *Cicuta*, Con., Croton-tig., *Kali-cb.*, KALI-CHLOR., KREOS., Laur., Led., Lyc., Mag-cb., Mag-mu., *Merc-cor.*, Merc-viv., Mezer, *Mur-ac.*, *Na-cb.*, NAT-MUR., *Nux*, *Paris*, Petrol., *Phos.*, Phos-ac., Puls., *Ran-bulb.*, RHUS, SEPIA, *Sil.*, Spong., STAPH., SUL., Tarax., Verat.

MOUTH, CORNERS of. *Ant-c.*, *Arsen.*, *Arum*, *Bell.*, *Bry.*, Calc., Calc-flu., *Caust.*, Cicut., *Cund.*, Graph., Hepar, *Iod.*, *Kali-bi.*, *Kreos.*, Mang., MERC., Na-carb., *Nat-mur.*, NIT-AC., Petrol., *Phos.*, *Sep.*, *Sil.*

MOUTH, ROOF of. See PALATE.

NOSE, ALÆ. *Aur.*, *Brom.*, Canth., Caust., Coccus-cact., Euphras., Mag-mu., Merc., *Mezer.*, *Na-mu.*, *Nux*, Phos., Plumb., Puls., Rhus., *Sil.*, Squil., Sul.

NOSE, BEGINNING in. Lyc., Merc-cyan.

NOSE, EXTENDING to. Kali-bi., Merc-cor., Merc-viv., Nit-ac.

NOSE, INTERNAL. Acon., Agar., Ailanth., All-cep., Alum., Am-cb., Am-mu., Anac., Ant-crud., Ant-tart., Apis, Arg-nit., Arn., *Arsen.*, ARUM., *Aur.*, Bary-cb., Bell., Borax, Brom., Bryon., *Calc.*, Camph., Canth., Caps, Caust., Cham., Cina, Clemat., Coccul., Coccus-cact., Colch., Con., Euphras., Flu-ac., Gamb., Graph., Hydras., Hyos., Ign., Iod., KALI-BI., *Kali-cb.*, *Kali-iod.*, Kali-nit., Kreos., Lach., *Lyc.*, Mar-v-t., *Merc-cor.*, Merc-j-ru., *Merc-viv.*, Mezer, Nat-ars., Nat-mur., NIT-AC., Nux, Petrol., Phos., Phytol., Plant., Podo., *Puls.*, Ran-bul., Rhus, Rumex, Sabadil., Secale, Senega, Sepia, SIL., Squil., Staph., Sticta., SUL., Therid., Thu., Verat.

NOSE, SEPTUM. Crot-tig., Hydrast., Kali-bi., Lyc., Merc., *Sil.*, *Sul.*

PALATE, ARCH of. Aloe, Am-caust., Apis, Bell., Berb., Kali-bi., Lyc., Merc-cyan., Merc-j-fl., Mezer., Mur-ac., Nit-ac., Tabac.

**REGION**—*Continued.*

PALATE, ARCH of, BEGINNING on. Apis.

PALATE, HARD. Acon., Apis, Arsen., Aur., *Bary-cb.*, BELL., Borax, Bry., *Calc.*, Camph., Canth., *Caps*, Carb-veg., Coccul., Colo., Crocus, Crot-tig., Euphor., Ign., Kali-cb., Lac-can., Meny., Merc., *Mezer.*, *Nit-ac.*, *Nux*, *Paris*, *Phos.*, Rhus, Sabad., Sang , Spig., Spong., *Squil.*, Staph., Sticta, *Sul.*, *Zinc.*

PALATE, SOFT. Acon., Am-caust., Arg-met., Arg-nit., *Arum*, *Aur.*, Bell., Dig., Dros., Iod., Kali-bi., Kali-nit., Lach., *Lac-can.*, *Merc-cor.*, Merc-j-fl., MERC-VIV., Mezer., *Mur-ac.*, *Nat-ars.*, *Na-mur.*, *Phos.*, *Phos-ac.*, Phytol., Ran-bulb., *Ran-scel.*, Sang., Sars., Sil., *Sticta.*, *Stram.*, Sul., Sul-ac., Zinc.

PALATE, SOFT, BEGINNING on. Apis., Arsen.

PALALE, SOFT, LEFT side of. Merc-cyan.

POSTERIOR, FAUCES. See FAUCES, POSTERIOR.

RIGHT SIDE. See SIDE, RIGHT.

SIDE, LEFT. Acon., Æscul., Ant-tart., Apis, Arum, Aur., *Bary-cb.*, *Bell.*, Bryon., Caps., Carb-an., *Caust.*, Colch., Crocus, Cup-met., Euphorb., Gels., *Graph.*, *Hep.*, Kali-bi., *Kali-cb.*, LACH., Lachnanth., *Lac-can.*, *Lyc.*, Marum-v-t., Meny., Merc-cyan, *Merc-j-ru.*, Mezer., Nit-ac., Nux-mos., *Nux-vom.*, Phos., Phytol., *Puls.*, Rhodo., *Rhus*, Sabina, Senega, *Sepia*, Sil., *Sul.*, Tarax., Thuj., Verat., Zinc.

SIDE, LEFT, BEGINNING on. LACH., Lac-can.

SIDE, RIGHT. Alum., *Am-cb.*, Arsen., Bell., Brom.. Bryon., Calc., Canth., Caps., *Carb-veg.*, Caust., China, Coloc., *Dros.*, *Flu-ac.*, Gels., Igna., *Kreos.*, *Lach.*, *Lac-can.*, LYC., *Mar-v-t.*, *Merc-j-fl.*, *Merc-viv.*, Na-mur., *Niccol.*, Nit-ac., Nux, Petrol., *Phytol.*, Plumb., Psor., Ran-bulb., Rhus, Sabad., Sal-acid, Sang., Sepia, Spig., Stan., Sul., Thu.

SIDE, RIGHT, BEGINNING on. *Lac-can.*, LYC., Merc-j-fl., Rhus.

TONGUE. Acon., Ailanth., Am-mu., *Apis*, *Arg-nit.*, *Arsen.*, ARUM., Bary-cb., *Bell.*, Borax, Bryon., Calc-ph., Canth., Caust., Cham., Chel., China, Dig., Hydras., Hyos., KALI-BI., Kali-bro., Kali-jod., *Lach.*, *Lyc.*, *Merc-cor.*, *Merc-j-fl.*, MERC-VIV., Mur-ac. *Nat-mur.*, NIT-AC., Nux, Opium, Phos., Plumb., Podo., Puls., Rhus, Sabad., Secale, Sep., Spig., Stan., Stram., SUL., Tarax., Verat., Verat-vir., Verbas., Zinc.

TONGUE, FORE PART. Bell., Merc-cor., Merc-viv., Sep., Sil.

TONGUE, FRÆNUM. Igna., Kali-cb.

TONGUE, MIDDLE. Acon., Arsen., Phos., Puls., Rhus, Verat.

TONGUE, ROOT of. Arum., Kali-bi., Nit-ac., *Phytol.*, Thuj.

TONGUE, SIDES of. Arsen., Canth., *Kali-bi.*, Lach., Merc-j-fl., Merc-viv., Phytol., Sul.

TONGUE, TIP of. Acon., Arg-nit., Arsen., Kali-cb., Merc-j-fl., Phytol., Puls., Rhus, Sep.

TONSILS. *Acon.*, *Æscul.*, Ailanth., Am-cb., Am-caust., *Am-mur.*, Apis, *Arsen.*, Bapt., BARY-CB. Bary-mur., BELL., Calc-ph., Canth., Crot-tig., Hep., Ign., Iod., *Kali-bi.*, LAC-CAN., LACH., LYC., Merc-cor., Merc-cyan., *Merc-j-fl.*, *Merc-j-ru.*, *Merc-viv.*, Mur-ac., NIT-AC., PHYTOL., Ran-bul., Sabad., Sul., Sul-ac.

**REGION**—*Continued.*

TONSILS, LEFT. See SIDE, LEFT.

TONSILS, RIGHT. See SIDE, RIGHT.

UVULA. *Apis*, Bell., Caps., Hyos., KALI-BI., Lac-can., Merc-cor., Merc-j-fl., Merc-j-ru., Merc-viv., Mur-ac., Nat-mur., Nit-ac., *Rhus*, Sul., Sul-ac.

VELUM. See PALATE, SOFT.

### CONCOMITANT.

APHONIA. See VOICE, LOSS OF.

BACK, PAIN IN, ON SWALLOWING. Kali-cb., Raphan., Rhus.

BREATHING, SUFFOCATIVE. See SUFFOCATION.

CERVICAL GLANDS. See GLANDS, CERVICAL.

CHEST, PAIN IN, ON SWALLOWING. Calc-ph.

CONVULSIONS, ON SWALLOWING. Lyc.

CROUP. See VOICE, CROUPY.

DYSPNŒA, VIOLENT. See SUFFOCATION.

ELONGATED UVULA. See UVULA, ELONGATED.

EPISTAXIS. See NOSE BLEEDING.

GLANDS, CERVICAL and submaxillary. Ailanth., Am-mur., Apis, *Arum.*, Aur., *Bary-cb.*, *Bell.*, *Calc.*, Carb-an., Carb-veg., Carbol-ac., Cham., Clem., Con., Cor-rub., Graph., Ign., Iod., Kali-bi., Kali-cb., Kali-iod., Lach., Lyc., Merc-cor., Merc-cyan., Merc-j-fl., Merc-j-ru., *Merc-viv.*, Nat-cb., Nit-ac., Psor., *Rhus*, Sil., Spig., Spong., *Staph.*, Sul., Sul-ac.

GLANDS, PAROTID. Aran., Arg-met., Arum, Bapt., *Bell.*, Brom., Bry., *Calc.*, Carb-an., Carb-veg., *Cham.*, Coccus-cact., *Con.*, Diosc., Form., Ign., *Kali-bi.*, Kali-cb., *Lach.*, *Lac-can.*, Merc-cyan., MERC-J-FL., Merc-j-ru., *Merc-viv.*, Plumb., RHUS, Sabad., Sep., Sil., Sul., Sul-ac., Sumb.

GLANDS, SALIVARY. Ambr., Bary-mu., Cham., China, Igna., Kali-nit., Merc-cor., Merc-j-fl., *Merc-viv.*, Thuj.

GLANDS, SUBMAXILLARY. See GLANDS, CERVICAL and SUBMAXILLARY.

HEAD DRAWN to one side. See TORTICOLIS.

HOARSENESS. See VOICE, HOARSE.

NECK DRAWN to one side. See TORTICOLIS.

NECK, STIFFNESS of. Am-mu., *Bell.*, *Bry.*, *Calc-ph.*, Chel., Crocus, Cycla., Dig., Fer-met., Glon., Hell., Lach., *Lachnan.*, Merc-j-fl., Merc-viv., Mezer., Nit-ac., Phytol., *Rhus*, Selen., Spig., *Spong.*, *Squill.*, Sul., Tabac.

NECK, STIFFNESS, LEFT side. *Bell.*, Brom., Carb-an., Chel., Coloc., Huron., Kreos., Laur., Lyc., Na-ars., Sil., Zingiber.

NECK, STIFFNESS, NAPE. Agar., Am-mur., Anac., *Ang.*, Ars., *Bary-cb.*, *Bell.*, Berb., *Bry.*, Calc., *Calc-ph.*, Camph., Canth., Caps., *Caust.*, Cor-rub., Dig., Dros., *Dulc.*, Glon., Graph., Guaic., *Hell.*, Ign., *Kali-cb.*, Lach., *Lyc.*, *Mag-cb.*, Mang., Meny., Merc-viv., Mezer., Nat-cb., Nat-mur., Nit-ac., Nux, Oleum-an., Phos., *Plat.*, Psor., Rat., *Rhod.*, *Rhus*, Secale, Selen., *Sep.*, *Sil.*, *Spong.*, *Squill.*, *Staph.*, *Sul.*, Thu., Verat., Zinc, Zingiber.

NECK, STIFFNESS, RIGHT side. *Caust.*, Elaps., Merc-j-fl., Nat-mur., Petrol., Sil.

### CONCOMITANT—*Continued.*

NECK, SWOLLEN. (Compare GLANDS.) Æscul., Ailanth., Arsen., Bary-cb., *Bell.*, Canth., Graph., Hydr-ac., Kali-iod., Kali-nit., Merc-sol., Rhus.

NECK, TENDERNESS of. See AGG. and AMEL. TOUCHING neck, aggr.

NOSE, BLEEDING. *Acon.*, Aloe, Ambr., Ant-c., *Arn.*, Arsen., Asar., Aur., Bary-cb., *Bell.*, Bism., Bovis., Bryon., Cactus., *Calc.*, Calc-ph., Canth., Caps., Carb-an., *Carb-veg.*, Cham., *China*, Colch., Con., *Crocus.*, *Crot-tig.*, Cup-met., Dig., Dros., Dulc., Elaps., Fer., Graph., *Ham.*, Hydras., Hyos., Igna., Ipec., Kali-cb., Kali-nit., Kreos., *Lach.*, Ledum, Lyc., Merc-cor., *Merc-viv.*, Mosch., Mur-ac., Nat-cb., Nat-mur., *Nit-ac.*, Nux-mos., Nux-vom., Paris, Petrol., *Phos.*, Phos-ac., Plat., *Puls.*, Ran-bulb., Rhus, Sabad., *Sabin.*, *Secale*, Sep., Sil., Squill., Staph., Stram., *Sul.*, Tereb., Thuj., Verat., Zinc.

PARALYSIS. Arn., Bary-cb., *Caust.*, *Coccus-cact.*, *Diphther.*, *Cup-met.*, Gels., *Nux*, Plumb., Rhus, Stan., Sul., *Syphil.*, Thu., Zinc.

PAROTID GLANDS. See GLANDS, PAROTID.

RESPIRATION, SUFFOCATIVE. See SUFFOCATION.

SALIVARY GLANDS. See GLANDS, SALIVARY.

SCARLET RASH, with. *Ailanth.*, Am-cb., Arsen., Arum, Bell., Carb-veg., Lach., Phytol., *Sul.*

SPASMS on swallowing. See CONVULSIONS.

STIFFNESS OF NECK. See NECK, STIFFNESS of.

STOMACH, PAIN IN, ON SWALLOWING. Calc-ph.

SUBMAXILLARY glands. See GLANDS, CERVICAL and SUBMAXILLARY.

SUFFOCATION threatened. Apis, Lach.

SWALLOWING, CONSTANT inclination. Æscul., Alum., Arsen., Arum, Bapt., *Bell.*, *Bry.*, Caust., Cistus, Cocc-cact., Con., Gels., Gratiol., Hydrophb., Kali-perm., Lach., Lact-acid., *Lac-can.*, Lyc., Merc-j-fl., *Merc-viv.*, Nat-sul., Nux-mos., Phytol., Plumb., Sabadil., Seneg., Sep., Staph., Tilia.

SWALLOWING, DIFFICULT: Am-caust., Arsen., Arum, Bary-cb., Bell., Bryon., Canth., Ign., Lach., *Lac-can.*, Lyc., Merc-j-fl., Merc-j-ru., Merc-viv., *Nit-ac.*, Phytol., Sul., Sul-ac.

SWALLOWING, DIFFICULT, FOOD (of). Bapt., Bary-cb., Rhus.

SWALLOWING, DIFFICULT, LIQUIDS (of). *Bell.*, *Canth.*, Igna., *Lach.*, Lyc., *Merc-cor.*, Stram., Sul-ac.

SWALLOWING IMPOSSIBLE. Acon., Ant-tart., Apis, Arum, Bell., Cicut., Gels., Hyos., Lyc., Mur-ac., Stram.

SWALLOWING, IMPOSSIBLE, SOLIDS (of). *Bapt.*, Bry., Ign., Sil.

SWALLOWING, RETURNS THROUGH NOSE. *Bell.*, Lach., Lac-can., *Lyc.*, Merc-viv., Nit-ac., Sul., Sul-ac.

TENDERNESS OF NECK. See AGGR. and AMEL. TOUCHING neck. Agg.

TORTICOLIS. Bell., Calc., Cina, LACHNAN., Lyc., Nux, Rhus, Sul.

UVULA, ELONGATED. Apis, Caps., Kali-bi., Lyc., Merc-cor., Merc-p-rub., Merc-viv., Nat-mur., Sul.

VOICE, CROUPY. Acon., Am-cb., Ant-tart., Asaf., Asar., Bell., *Brom.*, Cham., China, Dros., HEPAR, Iod., Kali-bi., LAC-CAN., *Phos.*, Samb., Sang., Spong., Sul-ac.

**CONCOMITANT**—*Continued.*

VOICE, DEEP. See VOICE, ROUGH.

VOICE, FEEBLE. See VOICE, WEAK.

VOICE, HIGH. (Squeaking.) Acon., Arsen., Cup-met., Dros., Lac-acid., Rumex, Stan., *Stram.*

VOICE, HOARSE. Acon., Alum., Ambr., Am-caust., Am-cb., Am-mur., Angus., Ant-tart., Apis, Arn., *Arsen.*, Bary-cb., *Bell.*, Berb., Bovis, Brom., *Bry.*, Calc., Calc-caust., Canth., Caps., Carb-an., *Carb-veg.*, *Caust.*, Cham., China, Cicut., Cina, Coccus-cact., Colch., Crot-hor., Cup-met., Dig., *Dros.*, Dulc., Fer., Gels., Graph., *Hepar*, Hydr-ac., Hyper., Iod., Kali-bi., Kali-cb., Kali-chl., Kali-nit., Kreos., *Lach.*, Lact., Laur., Led., Lyc., Mag-cb., Mag-mur., Mangan., Meny., Merc-cor., Merc-sulph., *Merc-viv.*, Mezer., Murex, Mur-ac., Nat-cb., Nat-mur., Niccol., Nit-ac., Nux-mos., Nux-vom., Opium, Oxal-ac., Paris, Petrol., *Phos.*, Phos-ac., Plumb., Puls., Rhod., Rhus, Sabad., Samb., Secale, Selen., Seneg., Sep., Sil., Spig., *Spong.*, Stan., Staph., Stront-c., *Sul.*, Sul-ac., Thu., Tilia, Verat., Verbas., Zinc.

VOICE, INDISTINCT. See VOICE, WEAK.

VOICE, LOSS of. Am-caust., Ant-c., Bell., *Brom.*, *Caust.*, Kali-iod., Lach., Merc-cyan., *Phos.*, Puls., Stram.

VOICE, NASAL. Aur., Bell., Bryon., Caust., Kali-iod., Lach., Merc-viv., Phos., Rumex, Sang., Sinap., Staph.

VOICE, ROUGH (bass). Alum., Ambr., Anac., Ant-c., *Bell.*, Brom., Bryon., *Carb-veg.*, Caust., China, Crotal-h., Dig., *Dros.*, Hepar, Hyos., Iod., Kali-bi., Nux, Paris, *Phos.*, Puls., Samb., Seneg., Spong., Stan., Sul., Verat., Verbas.

VOICE, WEAK. Am-caust., Ant-c., Ant-tart., Arsen., Bary-c., Bell., *Brom.*, Camp., Can-sat., Caust., Coccus-cact., Crot-hor., Gels., Hepar, Laches., Lyc., Phos., Stram.

WHISTLING SOUND IN TRACHEA. Merc-cyan.

WRY NECK. See TORTICOLIS.

Adjourned to 9.30 A. M.

---

*Fourth Day—Morning Session.*

FRIDAY, June 24th, 1892, 9.30 A. M.

The following were elected officers of the Association for the ensuing year: President, Edward Rushmore, M. D.; Vice-President, T. S. Hoyne, M. D.; Secretary, Samuel A. Kimball, M. D.; Corresponding Secretary, Samuel Long, M. D.; Treasurer, Franklin Powel, M. D.

Board of Censors: W. P. Wesselhœft, M. D., Chairman, E. T. Adams, M. D., H. C. Allen, M. D., B. Le Baron Baylies, M. D., A. R. Morgan, M. D.

Dr. A. B. Carr was appointed Necrologist.

The Chairmen for the Bureaus were appointed as follows:

(Dr. B. Fincke had been previously appointed Chairman of the Bureau of Homœopathic Philosophy.)

Bureau of Materia Medica, Dr. Geo. H. Clark; Bureau of Clinical Medicine, Dr. A. R. Morgan; Bureau of Obstetrics, Dr. Julia Morton Plummer; Bureau of Surgery, Dr. W. L. Reed.

The following resolution was presented by Dr. Custis and was adopted:

*Resolved:* That it is the sense of this meeting that we take part in the World's Congress of Physicians at Chicago, if practicable, and it is desired that we meet in the vicinity of Chicago.

